# Poplar Hospital for Accidents

**Published:** 1893-07-15

**Authors:** 


### POPLAR HOSPITAL FOR ACCIDENTS.

On Wednesday, July 5th, the thirty-eighth annual festival dinner in aid of the funds of this hospital was given at the Holborn Restaurant. The chair was taken by Mr. A. F. Hills, the President, and amongst those present were the Hon. Sydney Holland (chairman), Sir Donald Currie, Bart., M.P., the Rev. A. E. Dalton, Colonel Du Plat Taylor, C.B., the Rev. A. N. Campbell, Mr. E. Walmisley, and Mr. W. W. Aldridge. In proposing "Success to the Poplar Hospital," the President spoke of the special work which fell to the share of this institution, owing to its position in that part of London where the docks are situated. Accidents are many, and no accident case was ever refused either by day or night. The work went on all the year round, and the number of accidents reach an average of three for every working hour of every day. Those who benefited most from the help given by the hospital were amongst its most earnest supporters, and a sum of £500 was collected in pence and small donations from the very poorest. There was a sum of £3,000 to be cleared off at that dinner, and £3,400 had been received, of which it was intended to devote £2,250 to the building fund, and the rest would be used for the maintenance of the hospital. When the new buildings were opened in September, women as well as men would be received. The number of women employed in different industries in the East-end of London was very large, and it was desired to start the women's ward with as little delay as possible. The toast was responded to by the Hon. Sydney Holland, who, in an interesting speech, gave further details of the work of the hospital, concluding with an earnest appeal for sympathy and help.

